# Added Fructose in Non-Alcoholic Fatty Liver Disease and in Metabolic Syndrome: A Narrative Review

**DOI:** 10.3390/nu14061127

**Published:** 2022-03-08

**Authors:** Mattia Coronati, Francesco Baratta, Daniele Pastori, Domenico Ferro, Francesco Angelico, Maria Del Ben

**Affiliations:** 1 I Clinica Medica, Department of Clinical Internal, Anesthesiological and Cardiovascular Sciences, Sapienza University of Rome, 00161 Rome, Italy; coronati.1765853@studenti.uniroma1.it (M.C.); daniele.pastori@uniroma1.it (D.P.); domenico.ferro@uniroma1.it (D.F.); maria.delben@uniroma1.it (M.D.B.); 2 Department of Public Health and Infectious Diseases, Sapienza University of Rome, 00161 Rome, Italy; francesco.angelico@uniroma1.it

**Keywords:** NAFLD, metabolic syndrome, fructose, HFCS

## Abstract

Non-alcoholic fatty liver disease (NAFLD) represents the most common chronic liver disease and it is considered the hepatic manifestation of metabolic syndrome (MetS). Diet represents the key element in NAFLD and MetS treatment, but some nutrients could play a role in their pathophysiology. Among these, fructose added to foods via high fructose corn syrup (HFCS) and sucrose might participate in NAFLD and MetS onset and progression. Fructose induces de novo lipogenesis (DNL), endoplasmic reticulum stress and liver inflammation, promoting insulin resistance and dyslipidemia. Fructose also reduces fatty acids oxidation through the overproduction of malonyl CoA, favoring steatosis. Furthermore, recent studies suggest changes in intestinal permeability associated with fructose consumption that contribute to the risk of NAFLD and MetS. Finally, alterations in the hunger–satiety mechanism and in the synthesis of uric acid link the fructose intake to weight gain and hypertension, respectively. However, further studies are needed to better evaluate the causal relationship between fructose and metabolic diseases and to develop new therapeutic and preventive strategies against NAFLD and MetS.

## 1. Introduction

The relationship between non-alcoholic fatty liver disease (NAFLD) and metabolic disorders has been widely reported. Fatty liver might be regarded as the hepatic consequence of metabolic syndrome (MetS), a disease including central obesity, hyperglycemia, high blood pressure, hypertriglyceridemia, and low HDL cholesterol levels. Therefore, a strong reciprocal association between NAFLD and MetS has been proposed [1].

The global prevalence of NAFLD and MetS are 24% [2] and 25% [3], respectively. Both conditions are strongly associated with obesity and the risk of developing cardiovascular diseases, diabetes, chronic kidney disease, and non-alcoholic steatohepatitis (NASH) related liver complications [4,5,6,7,8].

Treatment and prevention of NAFLD and MetS are based on lifestyle intervention. Diet represents a key point for the improvement of MetS and lifestyle correction remains the only therapeutic approach for NAFLD [9]. Guidelines recommend the association of physical activity with caloric restriction, based on the Mediterranean diet, targeting a weight loss of at least 7%, to reduce liver steatosis [10].

Free sugars play a key role in the development of obesity, type 2 diabetes mellitus (DMT2), dental caries, metabolic syndrome (MetS), cardiovascular diseases and NAFLD. The World Health Organization suggests an intake of free sugars of less than 10% of the total energy intake [11]. To ensure this, many countries have applied different strategies including the application of a “sugar tax” on high sugar food and soft drinks [12,13].

Free sugars include all available carbohydrates as monosaccharides (glucose, fructose, and galactose) and disaccharides (lactose and sucrose). In particular, the role of fructose, added to foods during industrial processes via high fructose corn syrup (HFCS) and sucrose, is still debated. Although some studies highlight the association between fructose and cardiometabolic diseases, other studies deny this relationship which could be distorted by diet energy surplus.

The aim of this narrative review is to evaluate epidemiological, pathophysiological and clinical evidence on the association between the consumption of added fructose and NAFLD and MetS.

## 2. Natural and Industrial Sources of Fructose

Fructose is a glucose keto-hexose isomer with higher sweetness than sucrose. The food matrix modifies the effect of fructose on the human body [14,15,16]. Therefore, it is crucial to distinguish the food sources of this nutrient. Natural sources are represented by fruits, vegetables, and honey. Fructose may also be added to food through industrial processes using sucrose or HFCS [15]. The latter represents a valid alternative to sucrose in the food industry as it remains stable within acidic foods and drinks. The two HFCS types used on the market contain 42% and 55% fructose, respectively, and they are used for sodas and other sweetened beverages, candy, processed baked goods and condiments [17].

Furthermore, although the fructose molecule is the same in natural and industrial foods, the health effects appear to be different. This may be caused by the fact that the energy source in the processed product is more concentrated and available to the human body. Furthermore, important components such as fiber, vitamins, mineral salts, or antioxidants are not present in processed foods [18].

## 3. Metabolic Pathways Involving Fructose

**Figure 1:**
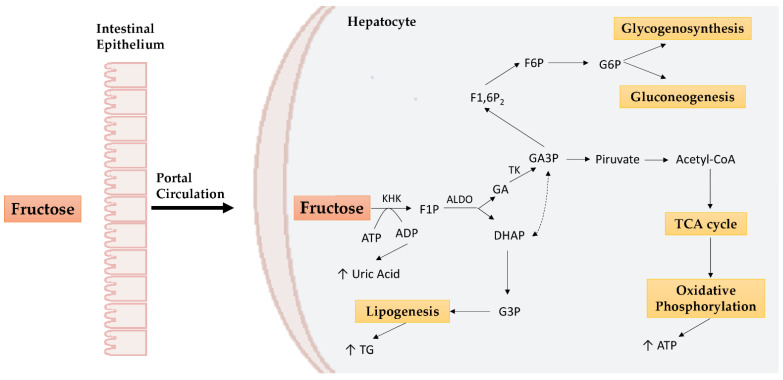
Hepatic fructose metabolism. In the liver, fructose undergoes KHK phosphorylation to obtain F1P. F1P obtained is metabolized to DHAP and GA by ALDO. DHAP is converted to G3P which enters the lipogenesis mechanisms. On the other hand, GA is converted to GA3P by TK. GA3P can promote lipogenesis after being converted to DHAP, but it can also generate pyruvate, involved, in turn, in the TCA cycle and oxidative phosphorylation to obtain ATP. GA3P is also a precursor of G6P used for gluconeogenesis or glycogenosynthesis. KHK: ketohexokinase; ALDO: aldolase; TK: triose kinase; ATP: adenosine triphosphate; ADP: adenosine diphosphate; F1P: fructose-1-phosphate; DHAP: dihydroxyacetone phosphate; G3P: glycerol 3-phosphate; GA: glyceraldehyde; GA3P: glyceraldehyde 3-phosphate; F1,6P_2_: fructose-1,6-bisphosphate; F6P: fructose-6-phosphate; G6P: glucose-6-phosphate; TG: triglycerides; TCA: tricarboxylic acid cycle.

Fructose is passively absorbed in the enterocytes by facilitative glucose transporter 5 (GLUT5) and to a lesser extent by facilitative glucose transporter 2 (GLUT2), which plays a major role in the liver. Most of the fructose, through the portal circulation, reaches the liver and undergoes fructolysis [19]. Fructokinase (KHK) and aldolase B (ALDO) enzymes induce the formation of glyceraldehyde (GA), which will enter the glycolytic or gluconeogenic pathway, and of dihydroxyacetone phosphate (DHAP), which will be used to form glycerol, essential for lipogenesis processes (Figure 1) [20]. In addition to KHK and ALDO, triose kinase (TK) plays a crucial role in fructose metabolism by promoting hepatic fat storage and preventing fructolytic substrates’ involvement in oxidative processes via other enzymes (e.g., aldehyde dehydrogenase) [21].

Unlike glucose, whose metabolism is finely regulated by phosphofructokinase, fructose does not undergo this restriction and fructolysis is potentially unlimited. The result is a large amount of substrate which is used in different metabolic pathways (glycolysis, glycogenesis, gluconeogenesis, lipogenesis, oxidative phosphorylation) according to cellular needs. As we will see below, this lack of regulation can promote metabolic alterations [22].

## 4. Fructose and Metabolic Diseases

The association between fructose consumption and metabolic diseases is debated. The strongest evidence was found with gout [23,24,25] and cardiovascular diseases [26,27,28]. Recently, attention was focused on the study of the pathophysiological mechanisms through which fructose could contribute to the development of MetS and NAFLD.

### 4.1. Fructose and NAFLD

The close relationship between NAFLD and lifestyle is widely proved. Several studies have reported the damaging or protective effect of different nutrients on liver health. NAFLD patients showed higher glucose and animal protein intake than controls who consumed more dietary fiber [29,30]. A high intake of saturated fatty acids (SFA), cholesterol and lower polyunsaturated fatty acids (PUFA), and vitamins consumption have also been documented in NASH patients [31]. In summary, a “Western diet”, characterized by a high intake of animal foods rich in SFA, trans fatty acids, refined cereals and soft drinks, represents a dietary model strongly associated with NAFLD, MetS, obesity and T2DM [32]. On the other hand, the Mediterranean diet, consisting of plant foods (fruit, vegetables and legumes), whole grains, olive oil and fish consumption, and the reduced intake of processed meat, dairy products, refined cereals and sweets, represents the best recommendation for the management and prevention of NAFLD and MetS [9,10,33]. For example, whole grains have a lower energy density than refined products and promote a greater sense of satiety and a lower overall energy intake [34]. They also influence the intestinal microbiota composition by reducing bacterial endotoxin absorption [35]. Both caloric surplus and bacterial endotoxin levels are positively associated with NAFLD and its complications [36,37].

A regular intake of ω-3 PUFAs (EPA and DHA) would seem to reduce hepatic lipogenesis and counteract insulin resistance and inflammation [38]. Moreover, ω-9 class monounsaturated fatty acids (MUFAs) have shown an improvement in the circulating lipid profile and in insulin resistance, thus suggesting a protective role against NAFLD [39]. Finally, a series of antioxidant compounds (alpha-tocopherol, carotenoids, polyphenols), contained mostly in fruits, vegetables, nuts and olive oil, have effects on the reduction in fatty liver [40].

Added fructose deregulates some physiological mechanisms and promotes liver fat accumulation. However, results are contradictory and might depend on the high energy intake induced by added fructose-rich diets. A meta-analysis revealed the ability of fructose to increase some NAFLD markers following a period of a high fructose high-calorie diet [41]. A 2016 study conducted in Germany on 143 patients showed that fructose consumption was higher in NAFLD patients than in healthy ones, but this difference was not significant when the calories consumed were normalized [29]. According to these data, caloric intake would seem to alter the effect of fructose on NAFLD.

However, Schwarz et al. showed that an intake of fructose equal to 25% of the caloric intake resulted in an increase in hepatic lipogenesis [42]. Furthermore, in patients with NAFLD, an increase in daily fructose intake was strongly associated with low levels of high-density lipoprotein (HDL), greater severity of fibrosis and liver inflammation [43]. Even in young people, an excess of added fructose led to alterations in the lipid profile in both healthy subjects and those with NAFLD [44]. Again, in a large longitudinal study conducted on 2600 participants, the increased consumption of sugary drinks was associated with fatty liver, regardless of possible confounding factors such as the total energy intake [45].

**Figure 2:**
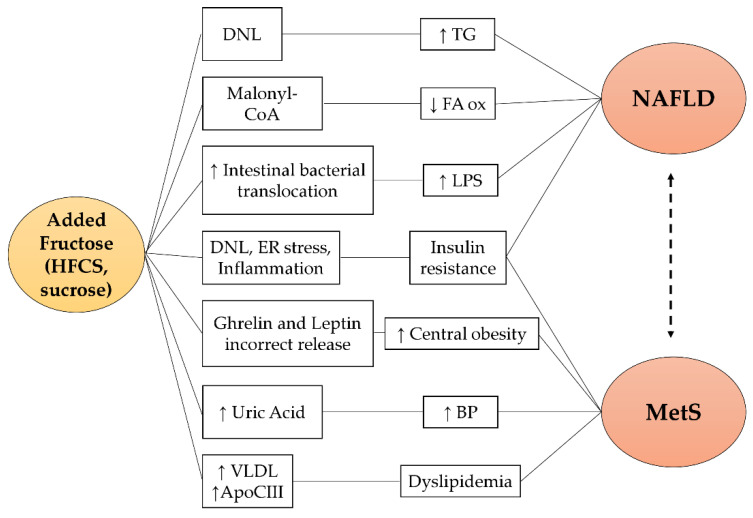
Fructose-related pathogenetic pathways in NAFLD and MetS. Fructose promotes NAFLD and MetS via different pathways: (1) it acts as a substrate and inducer of hepatic DNL resulting in steatosis and insulin resistance; (2) it is a precursor of malonyl CoA, an inhibitor of fatty acids oxidation; (3) it increases uric acid synthesis which is associated with hypertension; (4) it induces Apo CIII expression and the secretion of VLDL promoting dyslipidemia; (5) it favors intestinal bacterial translocation resulting in elevated serum LPS levels, closely associated with NAFLD; (6) it causes an incorrect regulation of the hunger–satiety mechanism which favors greater caloric intake and weight gain. HFCS: high fructose corn syrup; DNL: de novo lipogenesis; ER stress: endoplasmic reticulum stress; VLDL: very low-density lipoprotein; Apo CIII: apolipoprotein C-III; TG: triglycerides; FA ox: fatty acids oxidation; LPS: lipopolysaccharide; BP: blood pressure; NAFLD: non-alcoholic fatty liver disease; MetS: metabolic syndrome.

Fructose-related pathogenetic mechanisms for NAFLD are reported in Figure 2. A high fructose content diet induces a greater stimulation of de novo lipogenesis (DNL) due to the increased upregulation of transcription factors (SREBP1c and ChREBP) [46,47]. Concomitantly with the DNL induction, intermediate metabolites of fructolysis directly participate in the lipogenesis processes [20]. In addition, fructose promotes malonyl CoA production which inhibits fatty acids oxidation with consequent hepatic accumulation of triglycerides [48]. Finally, the chronic consumption of fructose alters the balance of the intestinal microbial flora and favors bacterial translocation processes, inducing a high circulating lipopolysaccharide (LPS) concentration [49]. LPS levels have been associated with the presence of NAFLD and the development of liver inflammation and fibrosis [37]. In particular, the increased expression and secretion of tumor necrosis factor (TNF), associated with high endotoxemia, stimulate SREBP1c activation, one of the protagonists of DNL [50]. In addition, in the intestinal lumen, fructose undergoes the gut microbiota action and is converted into acetate, which supplies lipogenic acetyl-CoA independently of the fructolytic metabolic processes [51].

Experimental studies demonstrated that eight weeks of a high fructose and sucrose diet induced liver fat accumulation and disease progression in a time of exposure dependent manner [52,53]. Similarly, an observational study performed in a group of young men showed the impaired synthesis of markers of fatty acids after the consumption of sugary drinks with a high fructose content. This effect did not occur for those who consumed sugary drinks rich in sucrose or glucose [54]. Finally, a randomized clinical trial conducted on NAFLD patients showed that 6 weeks of fructose dietary restriction led to a significant reduction in the hepatic lipid content [55].

### 4.2. Fructose and MetS

Metabolic syndrome (MetS) is a cluster of metabolic disorders, including central obesity, hyperglycemia, arterial hypertension, hypertriglyceridemia and low HDL-cholesterol, which affects about a quarter of the world’s population [3]. Several studies demonstrate a strong association between MetS and NAFLD, so that the latter has been considered the hepatic expression of MetS [56]. Insulin resistance, the main feature of MetS, is believed to play a central role in the early stages of fatty liver infiltration. However, it is still debated whether insulin resistance and hyperinsulinemia are components of MetS that promote fatty liver or whether NAFLD itself induces chronic hyperinsulinemia due to reduced insulin breakdown [1].

Fructose shows associations with MetS. Perez-Pozo et al. demonstrated the onset of MetS characteristics in healthy overweight adult men after a fructose daily intake in addition to the usual diet [57]. A cross-sectional study highlighted the link between fructose dietary consumption and MetS components, specifically hyperglycemia, central obesity and hypertriglyceridemia [58]. Considering this, added fructose could be considered a risk factor for MetS as well as for NAFLD.

Insulin resistance and blood glucose alteration have been related to the consumption of soft drinks containing fructose. Kimber et al. demonstrated that the prolonged intake of sugary drinks with fructose worsens insulin sensitivity and glucose tolerance [59]. Similar results were obtained by Aeberli et al. who showed a reduction in liver insulin sensitivity following a moderate fructose intake [60]. Nevertheless, different results were obtained from a 24-week RCT in which a diet with a low added fructose content did not improve insulin resistance. However, the study still showed an improvement in fasting blood glucose at the end of the low-fructose diet period [61]. The process underlying the alteration of glucose metabolism might be linked to a condition of hepatic insulin resistance arising from a prolonged exposure to fructose. In the liver, fructose supports de novo lipogenesis (DNL), induces endoplasmic reticulum stress and inflammation, leading to a reduction in hepatocytes’ insulin sensitivity [62,63].

In addition to these mechanisms, fructose also takes part in uric acid formation and hyperuricemia, also favored by insulin resistance. In fact, fructose increases uric acid both acutely, via the consumption of adenosine triphosphate (ATP) and the degradation of purines, and chronically by stimulating uric acid synthesis [64,65]. This is confirmed by several studies which show fructose’s effect on blood pressure. An observational study conducted in 4000 subjects showed that an intake of added fructose greater than 74 g/day (corresponding to 2.5 soft sugary drinks) was associated with higher values of blood pressure [66]. In 2010, a randomized clinical trial showed a significant increase in blood pressure in subjects who took 200 g/die of diluted fructose in water for two weeks. In particular, the treatment group showed a significant increase in uric acid. In the same work, reducing uric acid with allopurinol authors prevented blood pressure increase [57].

Sugary drinks and added fructose are also associated with dyslipidemia. In particular, 7 days of a high fructose content diet resulted in increased VLDL liver secretion [67]. Saito et al. found that the simultaneous ingestion of drinks containing fructose with a mixture of fats resulted in increased serum triglycerides [68]. Karen L. Teff et al. proved that fructose induced prolonged hypertriglyceridemia, differently from other sugars [69]. The mechanisms by which fructose promotes dyslipidemia involve DNL and the increased expression of Apo CIII. DNL, promoted by fructose through SREBP1c and ChREBP upregulation, is responsible for the increase in serum triglycerides [70]. Apo CIII also causes hypertriglyceridemia, supporting triglyceride mobilization during VLDL assembly and secretion. Finally, Apo CIII impairs the cholesterol efflux capacity of HDL-c particles, worsening the dyslipidemia [71].

Higher levels of added fructose intake were observed in obese patients [58]. In fact, several studies proved the contribution of soft drinks intake to weight gain [72,73,74,75]. Conversely, an interventional study with a low added fructose diet showed an improvement of central obesity [61]. The mechanisms underlying this are independent of the caloric diet surplus and involve insulin, leptin and ghrelin regulation [69]. Fructose intake determines a minimal insulin secretion which is insufficient to stimulate the adipocyte production of leptin. Lack of leptin synthesis prevents the triggering of satiety mechanisms by favoring further food intake [76]. Furthermore, fructose intake could contribute to the lack of ghrelin secretion inhibition. This is always caused by the reduced insulin secretion and glycemic stimulation that occur after fructose ingestion [69]. Lastly, the uncontrolled phosphorylation of fructose leads to an ATP deficit in the liver. In turn, ATP depletion promotes further energy intake through the diet, promoting weight gain [77].

## 5. Conclusions

The NAFLD and MetS epidemic worsens dangerously every year, along with other metabolic diseases such as diabetes and obesity. Currently, a lifestyle intervention is the best recommendation to improve liver steatosis and nutritional changes also represent the MetS treatment cornerstone.

Further intervention studies are necessary to confirm the causal participation of added fructose in NAFLD and MetS pathogenesis under real life conditions. In particular, it is advisable to avoid potentially confounding factors such as the diet calorie surplus and the excessive amount of fructose administered. This would allow the insertion of another important step in the understanding of the pathogenesis of NAFLD and MetS. However, the data presented in this review are robust and reliable and, in our opinion, a strong warning regarding high fructose food consumption, particularly sugary drinks, must be provided as part of the lifestyle modification for NAFLD patients.
